# Passive Remote Monitoring Technologies’ Influence on Home Care Clients’ Ability to Stay Home: Multiprovincial Randomized Controlled Trial

**DOI:** 10.2196/69107

**Published:** 2025-03-19

**Authors:** Lorie Donelle, Bradley Hiebert, Grace Warner, Michael Reid, Jennifer Reid, Salimah Shariff, Emily Richard, Sandra Regan, Lori Weeks, Kathleen Ledoux

**Affiliations:** 1 College of Nursing University of South Carolina Columbia, SC United States; 2 Arthur Labatt Family School of Nursing Western University London, ON Canada; 3 St Joseph's Health Care London London, ON Canada; 4 School of Occupational Therapy Dalhousie University Halifax, NS Canada; 5 Department of Health and Epidemiology Dalhousie University Halifax, NS Canada; 6 ICES Western London Health Sciences Centre Research Institute London, ON Canada; 7 Faculty of Nursing University of New Brunswick Moncton, NB Canada; 8 School of Health Administration Dalhousie University Halifax, NS Canada

**Keywords:** remote monitoring technology, home care, health service use, aging in place

## Abstract

**Background:**

Researchers in Nova Scotia and Ontario, Canada, implemented a passive remote monitoring (PRM) model of home care unique to their health system contexts. Each PRM model integrated tailored PRM devices (eg, motion sensors, cameras, and door alarms) into home care patients’ residences with the aim of linking patients, family and friend caregivers, and health care providers to support older adults’ aging in place.

**Objective:**

The purpose of this study was to examine the use of PRM technologies in the home to support older adults’ safe aging in place and avoidance or delay of higher levels of care.

**Methods:**

This multiprovincial pragmatic randomized controlled trial examined how PRM technologies support older adults to safely remain in their home and avoid or delay admission to higher levels of care. Pairs of home care patients and their family and friend caregivers were recruited in Ontario and Nova Scotia. Participant pairs were randomly assigned to one of two conditions: (1) standard home care (ie, control) or (2) standard home care plus study-provided PRM (ie, intervention). Participants provided their provincial health insurance numbers to link with provincial health administrative databases and identify if patients were admitted to higher levels of care after 1 year. Cox proportional hazards models were used to evaluate the primary outcome in each province.

**Results:**

In total, 313 patient-caregiver pairs were recruited: 174 pairs in Ontario (intervention: n=60; control: n=114) and 139 pairs in Nova Scotia (intervention: n=45; control: n=94). Results indicate PRM was associated with a nonsignificant 30% reduction in risk of patients being admitted to higher levels of care in Ontario (hazard ratio 0.7, 95% CI 0.3-1.4) and no reduction in risk in Nova Scotia (hazard ratio 1.1, 95% CI 0.3-3.7). Adjusting for patient sex had no impact on model estimates for either province.

**Conclusions:**

Limitations related, in part, to the impact of the COVID-19 pandemic may have contributed to the effectiveness of the intervention. While our study did not yield statistically significant results (*P*=.30 and *P*=.90) regarding the effectiveness of the PRM model in prolonging home stays, the observed trends suggest that technology-assisted aging in place may be a valuable goal for older adults. Further study is required to understand if longer follow-up time allows more effects of PRM on patients’ avoidance of higher levels of care to be detected.

**Trial Registration:**

ISRCTN ISRCTN79884651; https://www.isrctn.com/ISRCTN79884651

**International Registered Report Identifier (IRRID):**

RR2-10.2196/15027

## Introduction

Creative and effective ways to meet the health care needs of an aging population, often related to the high prevalence of chronic disease, are complicated by a strained health care work force, competing economic priorities, and evolving health information. Yet, these challenges create opportunities to reimagine how health care systems can provide Canadians with the right care, at the right time, in the right place. Although older adults may experience significant chronic disease that may challenge their ability to live independently, most older adults want to remain at home [1,2]. This sentiment was underscored by the findings of a recent survey where 85% of Canadian adults and 96% of Canadians aged ≥65 years reported that they would do everything they can to avoid institutionalization in a long-term care (LTC) setting [2,3]. Home care services that are appropriately managed and integrated into the health care system support older adults to successfully age at home, can improve the health and well-being of older adults and their families, and reduce costs of care in hospitals and LTC facilities [2,4]. However, in Canada, beyond the challenges previously noted, the provision of publicly funded home care services is limited and home care providers are increasingly unable to meet existing demands for home care service needs [2,4-6]. In fact, approximately 75% of home care support for older Canadians is provided *supposedly for free* by an unpaid family and friend caregiver [2,7], creating enhanced personal risk of physical health problems, stress, burnout, and depression [2,8].

A noted challenge of caring for older adults at home is providing suitable home care services and ensuring that older adults can safely follow established treatment plans. Common adverse events among older adults at home (eg, medication administration errors and falls) have been associated with increased use of health services, disability, and death [9,10]. Furthermore, many older adults have complex health needs, and the home care service gaps for older adults with complex care needs are further magnified by limited health human resources, access to partial home care services, a lack of direct support for or involvement of caregivers, and a lack of innovative strategies to expand home care [2,5]. These challenges highlight the urgency to develop more effective and respectful home care options for older adults.

A plausible intervention to address these home care challenges is the implementation of active and passive remote monitoring (PRM) technologies to support older adults to remain in their homes [11]. Active monitoring applications require individual participation, such as pushing a button on a wearable device (eg, pendant or bracelet), while passive monitoring technologies, such as motion sensors, do not require any action by the individual for the system to work. Remote monitoring is useful for tracking the behaviors of older adults with cognitive decline (eg, forgetting to take medications) and intervening quickly in the case of adverse events, such as falls. It also increases individuals’ confidence in their ability to care for themselves and live independently at home [11,12]. These technologies can also benefit older adults living at home and their caregivers by increasing communication and collaboration among people in their circle of care and by enabling big data analytics that can contribute to improving health care delivery practices [11,12]. While there is emerging evidence around the value of monitoring technologies to support older adults at home, there is limited insight into the outcomes of PRM, the preferences of older adults and their caregivers, and the costs and benefits associated with the wide variety of remote monitoring technologies available.

The purpose of this study was to examine the use of PRM technologies in the home to support older adults with complex care needs to safely remain in their home and avoid or delay admission to higher levels of care, such as long-term hospitalization and LTC. We evaluated a technology-enabled remote monitoring model of home care conceptually informed by the principles of person-centered care, family as a client, supported self-management, and stakeholder collaboration, to address gaps in home care for older adults. Supportive self-management speaks to the extended health care responsibilities taken up by older adults and their caregivers that create care work. Supportive self-management within home care would include both clinical (eg, medication administration, symptom monitoring, and care coordination) and nonclinical (eg, homemaking, meal preparation, supportive housing, daily check-ins, transportation, and a 24/7 helpline) funded services [13,14]. In this model of care, family was conceptualized as the patient or client receiving home care services *and* their caregiver. Typically, caregivers play an integral role as part of the care team to support older adults in the home; health care assessments remain largely focused on the needs of the older adult with relatively marginal inquiry into caregiver health and well-being [13,14]. The primary focus of this study was to investigate the impact of the PRM model of home care on older adult home care recipients with a secondary focus on assessing the impact of PRM on caregivers’ health and well-being. Reported here are our findings as to whether PRM along with usual home care supported older adults with complex care needs to safely remain in their home longer and delay or avoid admission to higher levels of care (ie, hospitalization and LTC) when compared with older adults receiving usual home care alone.

## Methods

### Overview

This study was informed by the findings of previous pilot studies conducted in the Canadian provinces of Alberta, British Columbia, New Brunswick, and Nova Scotia [15,16]. The primary outcome of this study was to support older adults with complex care needs to safely remain in their home and avoid or delay admission to higher levels of care, such as long-term hospitalization and LTC. This outcome was operationalized among complex care older adult home care recipients as the occurrence of and time to the following events: terminal admission to the hospital awaiting admission to LTC and direct admission to LTC. The secondary outcomes were an assessment of health service use and mortality rates of home care recipients within 1 year of trial enrollment. A complete description of the study’s methodology is reported elsewhere [17] and is summarized in the subsequent sections.

### Study Design

This study was an unblinded pragmatic randomized controlled trial (PRCT) [18,19] with 2 parallel arms in two Canadian provincial sites: (1) Central, Western, and Northern Zones in Nova Scotia and (2) the South West Region in Ontario. PRCTs are well-suited to supporting decision-making around complex interventions tested in the *real world* in comparison to usual forms of care [20,21]. As PRCTs are meant to capture real-world situations and the experiences of individuals, broad inclusion criteria developed in collaboration with regional health care partners were purposely selected for this study (see Textbox 1 for the full criteria). For this study, participants were recruited in home care client-caregiver dyads because the PRM requires that older adults have a family caregiver who is willing to receive system notifications. This PRCT is registered with the ISRCTN (registration 79884651).

**Participant eligibility.**

**Participant group and inclusion criteria (eligible if all criteria are met)**

- Home care clients
- Adult who is aged ≥65 years
- Requires home care and is at risk for higher levels of care as determined by the home care provider who makes these decisions
- Has a caregiver who is willing and able to receive the remote monitoring sensor notifications using a cell phone or a regular phone (ie, landline)
- Able to read and write in English or French
- Has the decisional capacity to consent or have a substitute decision-maker to consent for participation
- Caregivers
- Adult who is aged ≥18 years
- Is a caregiver to an adult who is aged ≥65 years that requires home care services and is assessed by home care service providers to be at risk for higher levels of care
- Can be a spouse, partner, child, sibling, other family relation, or friend who helps care for the patient at home
- Willing and able to receive the remote monitoring sensor notifications using a cell phone or a regular phone (ie, landline)
- Able to read and write in either English or French
- Has the decisional capacity to consent for participation

### Recruitment

The participant sample size was determined using information from one health region regarding the time to higher levels of care for older adults who previously required complex home care. The sample size calculation was based on the following criteria: total institutionalization proportion among controls being 0.41, and the proportion for the experimental participants being 0.27 (a 34% reduction compared with controls); a 10% attrition rate due to dropout or loss to follow-up; a power of 80%; and a statistical significance level of α=.05. This resulted in a total target sample size of 160 home care clients (and paired caregivers) for the intervention group and 320 home care clients (and paired caregivers) in the control group for a total study sample size of 480 home care clients and 480 caregivers across the Ontario and Nova Scotia study sites. The required sample was estimated based on a prospective test of independence (continuity-corrected chi-square statistic test) between the experimental and control groups using 2 controls per intervention case to increase the study’s power due to the cost of the intervention [22].

Participant recruitment occurred from April 2017 through January 2020 and was supported by the respective provincial regional authorities. Assessors and care coordinators employed at the health service provider that assesses and facilitates home care services in each region identified potential study participants (ie, clients) based on the study inclusion criteria (Textbox 1). The home care clients may have been on the assessors and care coordinators’ case load for up to 6 months before being contacted about the study. Care coordinators provided information about the study to potential study participants and their family and friend caregivers and requested permission to provide their names and contact information to the study research coordinator. Research staff contacted potential participants who agreed to have their contact information disclosed to arrange an appointment (in person or by telephone) to provide more information about the study. If both the home care client and family and friend caregiver met the eligibility criteria and verbally agreed to participate, the research staff scheduled an in-home meeting with the pair to obtain written consent and complete the baseline data collection forms (detailed in the *Data Collection* section).

### Randomization

With participants’ consent, baseline data were collected before random allocation into control or intervention groups. A block randomization process was used after every 3 pairs of participants were recruited [23] to randomly assign pairs in a 2:1 ratio to usual home care (ie, control group) or to usual home care plus study-provided PRM (ie, intervention group). This process was carried out independently in each province by members of the research team. Allocation bias was addressed through allocation concealment, and neither the home care case coordinator nor the researchers knew which group the participant would be assigned before baseline data collection. Following randomization research staff contacted each participant pair to inform them of their study arm. For intervention participants, research staff also forwarded their contact information to the technology provider to schedule an installation date.

### Control Group

Participants received their usual publicly funded home care services provided by their provincial or regional health care authority; some participants also purchased privately funded home care services to supplement the publicly funded services received. These services included home visits by assistive personnel for activities of daily living, nursing care, and other supports deemed necessary by the home care case coordinator or case manager. Home care assessments were conducted as usual by the regional authority case coordinator or case manager. Once the assessment was completed and care services were decided, services were provided by a contracted home care agency.

### Intervention Group

**Figure 1:**
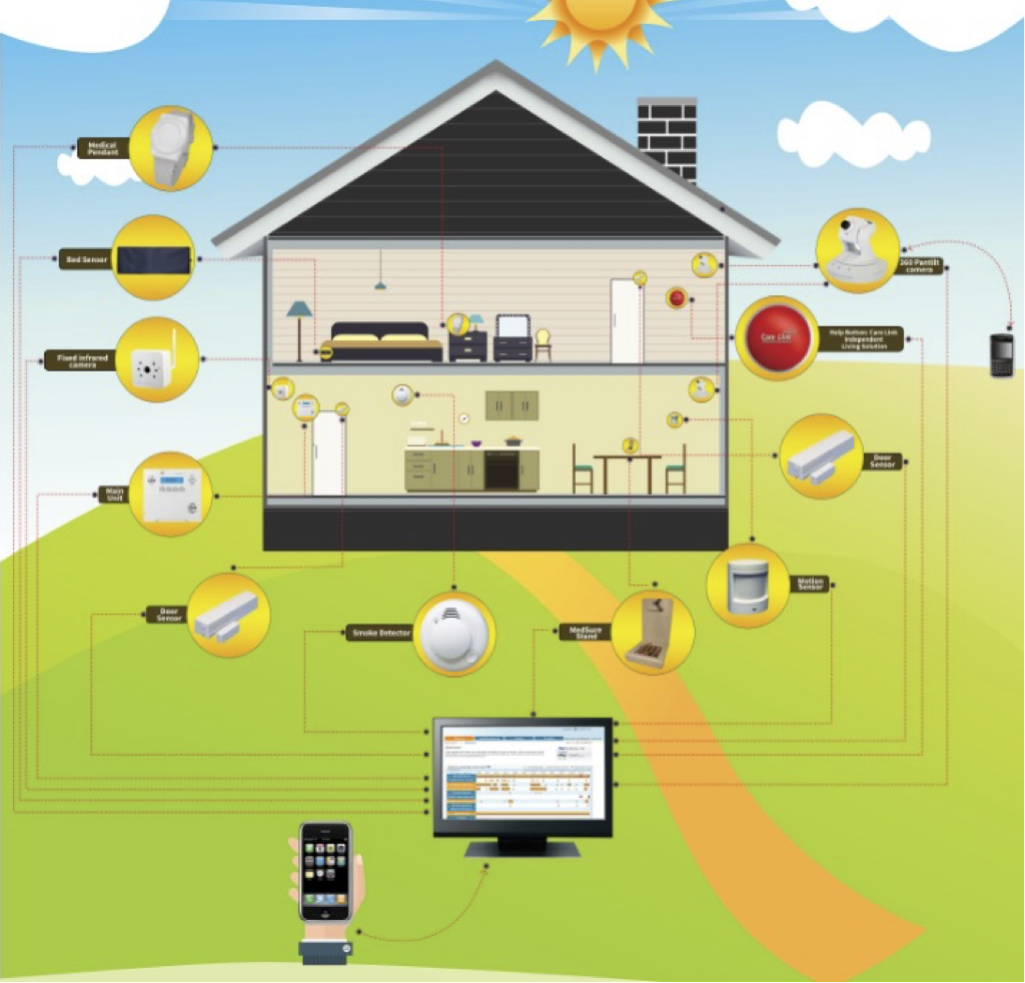
Example of a passive remote monitoring system setup.

Participants received their usual home care services (described earlier) and PRM systems provided and funded by the study technology partner. Once enrolled in the study, the technology partner visited the clients and their caregiver. Participants received written and verbal overviews of the PRM options. Each PRM system installed was tailored to meet the needs and preferences of both the home care client and their caregiver. PRM system options included a combination of long-life battery–powered sensors including motion sensors; Wi-Fi–enabled cameras; fall alert pendants or bracelets; contact sensors for internal or external doors, cupboards, and refrigerators; pressure mats that could be placed at the base of a toilet, under a mattress, or on a chair; and a medication sensor system. Each PRM system had a main panel that was connected to a phone jack installed in the home care client’s residence. The main panel received the signals from each sensor and sent them to a secured server to be transmitted back to the authorized recipient (eg, a caregiver) via a cellular signal or broadband module (Global System for Mobile Communications radio). The PRM systems only required internet access to transmit Wi-Fi-camera data. See Figure 1 for an example of how a PRM system used in this study could be set up.

The role of the caregiver was key to the successful implementation of the PRM intervention. In addition to an array of sensors, the technology partner, in consultation with the home care client and caregiver, configured each PRM system to provide home care clients and their caregivers with a range of alerts for atypical events tailored to their situation. Examples of alerts include reminders for clients to take their medication; the ability for caregivers to monitor medication use; home care client movement patterns within the home or when leaving the home; fall emergencies; abnormal eating patterns; and abnormal length of time in bed, on a chair, or in a toilet. Notifications of atypical events were sent to the caregiver via email, SMS text message, or phone call. Notably, notifications of home care clients’ atypical behaviors were not directly transmitted to a health care provider; rather, health care providers were notified at the discretion of the caregiver and in collaboration with the home care client, as appropriate. Possible caregiver actions based on notifications could include a telephone call to check on the client’s safety, deploying assistive home care supports, checking the video (if cameras are present), taking the client to seek medical attention, or emergency action such as calling an ambulance. The technology partner provided education (written and verbal) to the home care clients and caregivers about PRM sensors and the types of alerts; the technology provider technician communicated any changes to the sensor system within the home with the research staff.

The intervention was provided to participants for 12 months during the study. After this time, the home care client was transitioned to usual care if they did not wish to retain their PRM system at a reduced cost.

### Data Collection

#### Data Collection Window

Data collection aligned with the rolling recruitment of participants (see the *Recruitment* section) and lasted from April 2017 to February 2021. Paper format surveys comprised of standardized and researcher-developed tools were used to collect data from participant pairs three times over the course of a 12-month period: (1) at the point of recruitment when consent was obtained (ie, baseline), (2) 6 months following recruitment, and (3) 12 months following recruitment. Data were collected in person at the home care client’s home at baseline and by telephone or in person at the client’s home for the 6-month and 12-month follow-ups. Home care clients and caregivers were interviewed separately where possible.

#### Survey

Both home care clients and caregivers completed a survey consisting of validated questionnaires and researcher-developed questions to provide demographic information (eg, date of birth, education, sex, marital status, and household income); provincial health insurance numbers (used for data linkage to provincial administrative health databases); feelings of home care clients’ safety in their home; appraisals of home care quality; and satisfaction with and impact of PRM on both client and caregiver well-being (asked to the intervention group only). Additional researcher-developed items were asked of caregivers to understand their life satisfaction, self-rated mental health, and daily stress.

Home care clients completed standardized tools to assess their quality of life [24], Hospital Admission Risk Profile score, cognitive capacity (Mini-Mental State Exam) [25], and independent activities of daily living [26]. Caregivers also responded to standardized tools to assess their caregiving context, experiences, and information needs [27,28]; caregiver burden [29]; the home care client’s abilities to complete independent activities of daily living (a part of the Hospital Admission Risk Profile) [26]; positive aspects of caregiving [30]; presenteeism at work [31]; and the financial impact of caregiving [32].

#### Impact of the COVID-19 Pandemic on Data Collection

The onset of the COVID-19 pandemic response in March 2020 occurred during the data collection period. Research staff adhered to all public health guidelines for risk mitigation and collected data entirely by phone during this period. Changes to health care service delivery models and stay-at-home orders in Ontario and Nova Scotia altered how these participants (1) could access and use health care services and (2) interacted with their participant partner (ie, the other half of the client-caregiver dyad). Participants’ adherence to public health stay-at-home orders resulted in behaviors (eg, hesitancy to engage with acute and LTC services) that completely conflicted with and was contrary to our research outcome creating significant bias within the proportion of participants’ data collected after March 2020. In consultation with epidemiologists and statisticians, a decision was made to control for the effects of the pandemic response, and participant pairs were only included for analysis if all study activities were completed before March 2020.

### Administrative Data Linkage

#### Overview

Participants were enrolled between April 11, 2017, and January 15, 2020, in Ontario and between November 29, 2017, and December 16, 2019, in Nova Scotia. To determine the impact of the intervention on the primary study outcomes, the data collected by the research team (herein referred to as the PRCT database) was linked to health administrative databases held at the Institute for Clinical Evaluative Sciences (ICES; Ontario) and Health Data Nova Scotia (HDNS; Nova Scotia).

ICES is an independent, nonprofit research institute funded by an annual grant from the Ontario Ministry of Health and the Ministry of Long-Term Care. As a prescribed entity under Ontario’s privacy legislation, ICES is authorized to collect and use health care data for the purposes of health system analysis, evaluation, and decision support. Secure access to these data is governed by policies and procedures that are approved by the Information and Privacy Commissioner of Ontario.

HDNS is a similar institution to ICES. It is a data repository based in the Faculty of Medicine, Department of Community Health and Epidemiology at Dalhousie University, that is focused on supporting data-driven research for researchers in Nova Scotia. HDNS facilitates research and innovation in Nova Scotia by providing access to linkable administrative health data and analysis for research and health service assessment purposes in a secure, controlled environment, while respecting the privacy and confidentiality of Nova Scotians.

#### Data Sources

We used the PRCT database to identify patient and caregiver participants, including measures of risk that the patient had to alternate levels of care and hospital admission and measures of burden on caregivers. Health administrative databases in both Ontario and Nova Scotia were used to determine other baseline characteristics and outcomes and identify participant data, as follows:

1. Demographic and geographic information as recorded in administrative data
2. Preexisting comorbidities (using validated algorithms)
3. Previous emergency department visits
4. Previous surgeries
5. Previous outpatient, primary care, and specialist visits and follow-up LTC claims
6. Previous prescription use and follow-up LTC
7. Previous home care services
8. Previous and follow-up hospital admissions
9. International classification of diseases diagnosis codes—version 10 and Ontario health insurance plan diagnosis codes.

In Ontario, datasets were linked using unique encoded identifiers and analyzed at ICES. In Nova Scotia, the databases were matched whenever possible. When databases could not be matched, HDNS used alternate databases with similar data to approximate what was done in Ontario. For a complete listing of all databases used, see Table 1.

**Table 1:** Databases used by the Institute for Clinical Evaluative Sciences (ICES) and Health Data Nova Scotia (HDNS) to support analysis.

Characteristics	ICES databases (Ontario)	HDNS databases (Nova Scotia)
Demographic and geographic information	The registered persons database and PCCF^a^	Insured patient registry (MASTER) Postal code conversion file (PCCF+)
Preexisting comorbidities using validated algorithms	Ontario Diabetes Dataset, Chronic Obstructive Pulmonary Disease, congestive heart failure, Ontario hypertension dataset (HYPER), Ontario asthma dataset (ASTHMA), Ontario dementia database (DEMENTIA)	MSI physician’s billings (MED)
Emergency department visits	NACRS^b^	NACRS
Surgeries	Same Day Surgery database	—^c^
Outpatient, primary care, and specialist visits and follow-up long-term care claims	OHIP^d^ claims	MSI physician’s billings (MED) Eligibility group (EGROUP)
Prescription use and follow-up long-term care	Ontario Drug Benefit Claims and Drugs list	Nova Scotia Drug Information System
Home care services	Home Care Database	Nova Scotia Health Department of Seniors and Long Term Care
Hospital admissions	DAD^e^	DAD
Descriptions for international classification of diseases diagnosis codes—version 10 and OHIP diagnosis codes	REF^f^	REF

^a^PCCF: postal code conversion file.

^b^NACRS: National Ambulatory Care Reporting System.

^c^Not available.

^d^OHIP: Ontario health insurance plan.

^e^DAD: Discharge Abstract Database.

^f^REF: reference files.

### Primary Outcome

The primary outcome of interest was to support older adults with complex care needs to safely remain in their home and avoid or delay admission to higher levels of care, such as long-term hospitalization and LTC. The primary outcome was defined as time to a client’s inability to return home given the need for LTC or hospital admission under an alternative level of care designation within 1 year of enrollment in the trial. This was ultimately determined by the most responsible provider. The secondary outcome of health service use and mortality was assessed to confirm similar mortality rates within 1 year of enrollment in the trial. Clients were followed up to 1 year until February 28, 2020, to control for the impact of the COVID-19 pandemic. Participants enrolled before February 28, 2019, were included to ensure 1 year of follow-up before the COVID-19 pandemic.

### Data Analysis

Baseline characteristics were summarized using descriptive statistics: continuous variables as means and SDs and categorical variables as proportions. Baseline characteristics between the control and intervention participants were examined to verify successful randomization using standardized differences that when greater than 0.1, indicates a potentially meaningful difference [33]. In accordance with ICES and HDNS privacy policies, cell sizes less than or equal to 5 were not reported.

A Cox proportional hazards model was used to evaluate our primary outcome of unable to return home, censoring on mortality and 1 year after enrollment, and our secondary outcome of mortality, only censoring on 1 year after enrollment. Hazard ratios (HRs) with 95% CI were reported. Proportional hazards were assessed using interaction with time terms, and no violations were observed.

Subgroup analyses compared health care use 1 year before and after enrollment for home care clients to determine what effect the intervention had on participants’ use of the health care system. For the subgroup analysis, a paired analysis was conducted using the McNemar test for proportions and the Wilcoxon signed rank test for means to compare differences in health care use 1 year before and after enrollment in both patients (Ontario: n=108; Nova Scotia: n=105) and caregivers (Ontario: n=104; Nova Scotia: n=101). All analyses were conducted using SAS (version 9.4; SAS Institute). A statistical significance was defined as a 2-tailed α value less than .05.

### Ethical Considerations

This study was approved by the Research Ethics Board at Western University (registration IRB 00000940) and at Nova Scotia Health (registration IRB 102203). All participants provided written informed consent and could withdraw from the study at any time without penalty. All data were deidentified and assigned a study ID number to support data linkages throughout data collection and analyses. Participants received no compensation to complete the study; intervention group participants received the PRM systems at no personal expense.

## Results

Due to challenges joining administrative patient data—such as data collected as part of this study—across provincial jurisdictions, a single analysis was not possible. The following sections present the results of the separate analyses conducted by ICES in Ontario and HDNS in Nova Scotia to address this study’s research question.

### Participant Characteristics

#### Ontario Cohort

**Figure 2:**
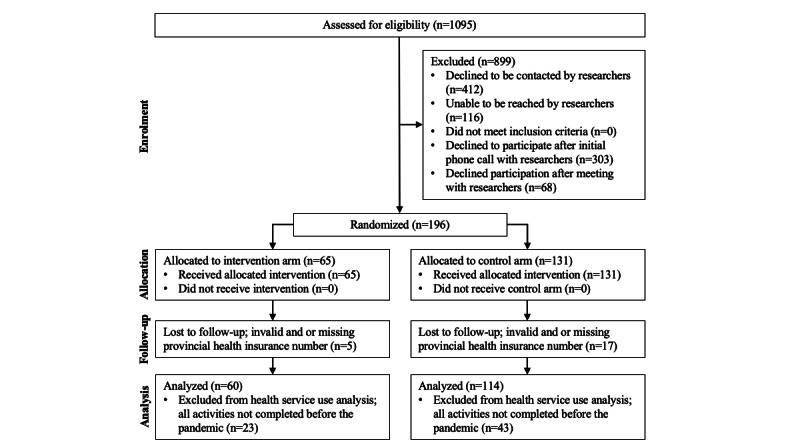
Ontario home care client cohort randomization and analysis flow diagram.

The total Ontario cohort comprised 339 participants (home care clients: n=174; caregivers: n=165). In total, 196 home care clients were enrolled in Ontario from April 11, 2017, to January 15, 2020, of which 22 were excluded due to invalid or missing health card numbers, leaving 174 home care clients to be included in analysis. We were able to link 165 caregivers to the home care clients. In Ontario, 116 participants (home care clients: n=60; caregivers: n=56) included in this analysis received the study-provided PRM intervention plus their normal home care and 223 participants (home care clients: n=114; caregivers: n=109) received their normal home care without PRM. Figure 2 provides the randomization and analysis study flow diagram for the Ontario home care client cohort.

#### Nova Scotia Cohort

**Figure 3:**
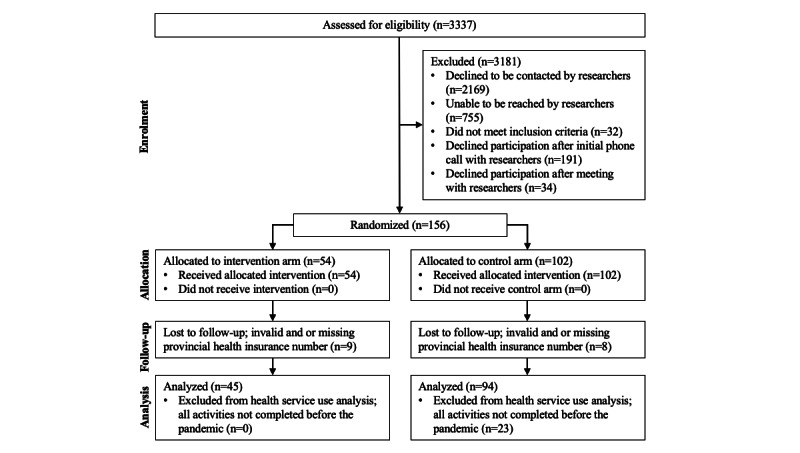
Nova Scotia home care client cohort randomization and analysis flow diagram.

The total Nova Scotia cohort comprised 273 participants (home care clients: n=139; caregivers: n=134). In total, 156 home care clients were enrolled in Nova Scotia from November 29, 2017, to December 16, 2019, of which 17 were excluded due to invalid or missing health card numbers, leaving 139 home care clients to be included in the analysis. We were able to link 134 caregivers to the home care clients. In Nova Scotia, 92 participants (home care clients: n=45; caregivers: n=47) included in this analysis received the study-provided PRM intervention plus their normal home care and 181 participants (home care clients: n=94; caregivers: n=87) received their normal home care without PRM. Figure 3 provides the randomization and analysis study flow diagram for the Nova Scotia home care client cohort.

### Home Care Clients

#### Ontario Home Care Clients

In Ontario, there were no significant differences between home care clients in intervention and control groups at baseline on any of the demographic characteristics evaluated (all *P*>.05). At baseline, Ontario home care clients were on average aged 78 (SD 6.9) years. There was a higher proportion of male participants in the intervention group (40/60, 67%) compared with the control group (57/114, 50%). Most home care clients (125/174, 73%) resided in midsize urban centers. Approximately 20% (35/174) of home care clients resided in the lowest-income neighborhoods (quintile 1), while about 15% (26/174) resided in the highest-income neighborhoods. Most Ontario home care clients (109/174, 63%) lived with their spouse. Nearly all Ontario home care clients (>106/174, >90%) were at medium or high risk of hospitalization. Ontario home care clients had a variety of chronic disease comorbidities, most notably hypertension (144/174, approximately 83%), diabetes (95/174, approximately 55%), and dementia (67/174, approximately 40%). Complete home care client baseline characteristics are presented in Table 2.

**Table 2:** Ontario and Nova Scotia home care client baseline characteristics.

Characteristics			Ontario				Nova Scotia		
			Control (n=114)		Intervention (n=60)		Control (n=94)		Intervention (n=45)
Age (y), mean (SD)			77.9 (6.9)		78.1 (7.0)		80.1 (7.9)		81.5 (7.3)
**Sex, n (%)**									
	Female	57 (50)		20 (33.3)		62 (66)		33 (73.3)	
	Male	57 (50)		40 (66.7)		32 (34)		12 (26.7)	
**Level of rurality, n (%)**									
	Large CMA^a^ (≥500,000)	0 (0)		0 (0)		0 (0)		£5^b^	
	Midsize CMA (100,000-500,000)	80 (70.2)		45 (75)		66 (70.2)		35 (77.8)	
	Small CMA (10,000-100,000)	24 (21.1)		9 (15)		13 (13.8)		6 (13.3)	
	Non-CMA	10 (8.8)		6 (10)		15 (16)		£5	
**Neighborhood-level income quintile, n (%)**									
	Quintile 1 or missing	24 (21.1)		14 (23.3)		16 (17)		9 (20)	
	Quintile 2	20 (17.5)		15 (25)		29 (30.9)		17 (37.8)	
	Quintile 3	28 (24.6)		9 (15)		19 (20.2)		6 (13.3)	
	Quintile 4	23 (20.2)		10 (16.7)		13 (13.8)		7 (15.6)	
	Quintile 5	14 (12.3)		12 (20)		17 (18.1)		6 (13.3)	
**Living arrangements, n (%)**									
	Alone	≤5		0 (0)		56 (59.6)		29 (64.4)	
	With spouse	71 (62.3)		38 (63.3)		12 (12.8)		≤5	
	With children	0 (0)		0 (0)		0 (0)		0 (0)	
	Other	≤45		22 (36.7)		0 (0)		≤5	
	Missing	0 (0)		0 (0)		26 (27.6)		8 (18)	
**HARP^c^, n (%)**									
	Low risk	≤5		≤5		8 (8.5)		≤5	
	Medium risk	73 (64)		33 (55)		43 (45.7)		≤20	
	High risk	≤40		≤30		40 (42.6)		23 (51.1)	
**Comorbidities (prevalence in sample), n (%)**									
	Hypertension	95 (83.3)		49 (81.7)		31 (33)		21 (46.7)	
	Diabetes	61 (53.5)		34 (56.7)		29 (30.9)		12 (26.7)	
	Dementia	42 (36.8)		25 (41.7)		31 (33)		12 (26.7)	
	COPD^d^	40 (35.1)		18 (30)		15 (16)		10 (22.2)	
	CHF^e^	30 (26.3)		16 (26.7)		7 (7.4)		6 (13.3)	
	Asthma	25 (21.9)		10 (16.7)		≤5		≤5	
	AMI^f^	6 (5.3)		≤5		≤5		≤5	

^a^CMA: census metropolitan areas.

^b^Small cell sizes (ie, n£5) suppressed following the Institute for Clinical Evaluative Sciences and Health Data Nova Scotia reporting guidelines for maintaining participant confidentiality.

^c^HARP: Hospital Admission Risk Profile.

^d^COPD: chronic obstructive pulmonary disease.

^e^CHF: congestive heart failure.

^f^AMI: acute myocardial infarction.

#### Nova Scotia Home Care Clients

In Nova Scotia, there were no significant differences between home care clients in intervention and control groups at baseline on any of the demographic characteristics evaluated (all *P*>.05). In Nova Scotia, home care clients were on average 81 (SD 7.5) years of age. At baseline, there was a higher proportion of male participants in the control group (32/94, 34%) compared with the intervention group (12/45, 27%). Most Nova Scotia home care clients (101/139, 75%) resided in midsize urban centers. Approximately 20% (25/139) resided in the lowest-income neighborhoods, while approximately 15% (23/139) resided in the highest-income neighborhoods. The majority of Nova Scotia home care clients (85/139, approximately 60%) lived alone. Nearly all home care clients (>114/139, >90%) were at a medium or high risk of hospitalization. The Nova Scotia home care clients also had a variety of chronic disease comorbidities, most notably hypertension (52/139, approximately 40%), dementia (43/139, approximately 30%), and diabetes (41/139, approximately 30%). Complete home care client baseline characteristics are presented in Table 2.

### Family and Friend Caregivers

#### Ontario Caregivers

In Ontario, there were no significant differences between intervention and control caregivers at baseline for any of the characteristics evaluated (all *P*>.05). Ontario caregivers were on average 67 (SD 12.4) years of age. There was a higher proportion of male participants in the control group (39/109, 36%) compared with the intervention group (13/56, 23%). Most caregivers (116/165, 70%) resided in midsize urban centers. Approximately 20% (32/165) resided in the lowest-income neighborhoods, while about 17.6% (29/165) resided in the highest-income neighborhoods. About 40% (<55/165) of Ontario caregivers attained a postsecondary education. Just over half of the caregivers in both groups (99/165, 60%) were retired, with 10% (12/109) of control and 25% (15/56) of intervention caregivers reporting full-time employment. Approximately 66% (114/165) of caregivers were participating in the study with their spouse or partner, and over 80% (144/165) of caregivers lived with the home care client they supported. Approximately 33% (58/165) indicated they would consider moving the home care client to a LTC facility. Ontario caregivers also had a variety of chronic disease comorbidities, most notably hypertension (control: 56/109, 51%; intervention: 37/56, 66%), diabetes (32/165, 20%), and chronic obstructive pulmonary disease (29/165, 18%). Ontario caregivers provided an average of 109 to 119 hours of care or support each week. Scores on the Zarit Burden Interview suggest that caregivers in both groups were similarly burdened by caregiving responsibilities and that such levels of caregiver burden approached high levels of burden. Complete caregiver baseline characteristics are presented in Table 3.

**Table 3:** Ontario and Nova Scotia family and friend caregiver baseline characteristics.

Characteristics			Ontario				Nova Scotia		
			Control (n=109)		Intervention (n=56)		Control (n=87)		Intervention (n=47)
Age (y), mean (SD)			66.8 (12.2)		67.3 (12.6)		65.4 (10.9)		60.6 (12.1)
Hours spent providing care or support for home care client per week, mean (SD)			109.0 (68.8)		119.0 (67.9)		80.9 (69.3)		80.6 (71.4)
Caregiver burden^a^, mean (SD)			17.8 (9.7)		16.8 (8.4)		17.0 (9.3)		18.3 (11.7)
**Sex, n (%)^b^**									
	Female	70 (64.2)		43 (76.8)		55 (63.2)		33 (70.2)	
	Male	39 (35.8)		13 (23.2)		32 (36.8)		14 (29.8)	
**Level of rurality, n (%)^b^**									
	Large CMA^c^ (≥500,000)	≤5		0 (0)		0 (0)		0 (0)	
	Midsize CMA (100,000-500,000)	75 (68.8)		41 (73.2)		61 (70.1)		38 (80.9)	
	Small CMA (10,000-100,000)	23 (21.1)		8 (14.3)		13 (14.9)		6 (12.8)	
	Non-CMA	≤17		7 (12.5)		13 (14.9)		≤5	
	Missing	≤5		0 (0)		0 (0)		≤5	
**Neighborhood-level income quintile, n (%)^b^**									
	Quintile 1	22 (20.2)		10 (17.9)		15 (17.2)		6 (12.8)	
	Quintile 2	18 (16.5)		19 (33.9)		20 (23)		12 (25.5)	
	Quintile 3	≤40		7 (12.5)		16 (18.4)		13 (27.7)	
	Quintile 4	24 (22)		7 (12.5)		15 (17.2)		7 (14.9)	
	Quintile 5	16 (14.7)		13 (23.2)		21 (24.1)		9 (19.1)	
	Missing	≤5		0 (0)		0 (0)		0 (0)	
**Self-reported highest level of education, n (%)^b^**									
	Elementary school	21 (18.4)		9 (15)		10 (11.5)		7 (14.9)	
	High school	38 (33.3)		17 (28.3)		25 (28.7)		18 (38.3)	
	College, CEGEP^d^, or university	40 (35.1)		28 (46.7)		42 (48.3)		15 (31.9)	
	Postgraduate degree	12 (10.5)		≤5		10 (11.5)		≤5	
	Other, declined, or N/A^e^	≤5		≤5		0 (0)		≤5	
	Missing	≤5		≤5		0 (0)		0 (0)	
**Self-reported employment status, n (%)^b^**									
	Full time	12 (10.5)		15 (25)		23 (26.4)		13 (27.7)	
	Part time	20 (17.5)		≤5		10 (11.5)		9 (19.1)	
	Leave of absence	≤5		0 (0)		≤5		≤5	
	Retired	65 (57)		34 (56.7)		48 (55.2)		14 (29.8)	
	Not employed	14 (12.3)		6 (10)		≤5		10 (21.3)	
	Declined or N/A	0 (0)		0 (0)		0 (0)		≤5	
	Missing	≤5		≤5		≤5		≤5	
**Relationship to home care client, n (%)^b^**									
	Spouse or partner	73 (64)		41 (68.3)		34 (39.1)		14 (29.8)	
	Adult child	25 (21.9)		16 (26.7)		35 (40.2)		24 (51.1)	
	Parent	6 (5.3)		0 (0)		≤5		≤5	
	Other, declined, or N/A	≤15		≤5		17 (19.5)		8 (17)	
	Missing	≤5		≤5		≤5		≤5	
**Living with home care client, n (%^b^)**									
	Yes	95 (83.3)		49 (81.7)		59 (67.8)		30 (63.8)	
**Would consider moving home care client to long-term care facility, n (%)^b^**									
	No	≤80		≤50		64 (73.6)		32 (68.1)	
	Yes	38 (33.3)		20 (33.3)		22 (25.3)		14 (29.8)	
	Declined or N/A	≤5		0 (0)		≤5		≤5	
	Missing	≤5		≤5		≤5		≤5	
**Comorbidities, n (%)^b^**									
	Hypertension	56 (51.4)		37 (66.1)		34 (39.1)		13 (27.7)	
	Diabetes	19 (17.4)		13 (23.2)		22 (25.3)		7 (14.9)	
	COPD^f^	17 (15.6)		12 (21.4)		≤5		≤5	
	CHF^g^	6 (5.5)		≤5		0 (0)		0 (0)	
	Asthma	10 (9.2)		7 (12.5)		≤5		≤5	
	Dementia	≤5		0 (0)		≤5		0 (0)	
	AMI^h^	≤5		≤5		≤5		0 (0)	

^a^Caregiver burden assessed using the 12-item Zarit Burden Interview [29]. Scores range from 0 to 48, with higher scores reflecting higher levels of caregiver burden and scores of 17 and higher indicating a high level of caregiver burden.

^b^Small cell sizes (ie, n£5) suppressed following the Institute for Clinical Evaluative Sciences and Health Data Nova Scotia reporting guidelines for maintaining participant confidentiality.

^c^CMA: census metropolitan area.

^d^CGEP: collège d'enseignement général et professionnel.

^e^N/A: not applicable.

^f^COPD: Chronic Obstructive Pulmonary Disease.

^g^CHF: congestive heart failure.

^h^AMI: acute myocardial infarction.

#### Nova Scotia Caregivers

In Nova Scotia, there were no significant differences between intervention and control caregivers at baseline for any of the characteristics evaluated (all *P*>.05). On average, caregivers were aged 63 (SD 11.6) years. Approximately 35% (46/134) of Nova Scotia caregivers were male. Most caregivers (99/134, 75%) resided in midsize urban centers. Approximately 15% (21/134) of Nova Scotia caregivers resided in the lowest-income neighborhoods, while about 20% (30/134) resided in the highest-income neighborhoods. Postsecondary school was the highest level of education for 48% (42/87) of control caregivers and 32% (15/47) of intervention caregivers. Less than 30% (36/134) of Nova Scotia caregivers were employed full time; approximately 50% (48/87) of control caregivers and 30% (14/47) of intervention caregivers were retired. Approximately 35% (48/134) were participating with their spouse or partner, while approximately 45% (59/134) were participating with their parent. Approximately 66% (99/134) of Nova Scotia caregivers lived with the home care client they supported. Approximately 25% (36/134) of caregivers indicated they would consider moving the home care client to an LTC facility. Nova Scotia caregivers also had a variety of chronic disease comorbidities, most notably hypertension (control: 34/87, 39%; intervention: 13/47, 28%) and diabetes (control: 22/87, 25%; intervention: 7/47, 15%). Nova Scotia caregivers provided an average of approximately 81 hours of care or support each week. Scores on the Zarit Burden Interview suggests that caregivers in both groups were similarly burdened by caregiving responsibilities and that such levels of caregiver burden constituted high levels of burden. Complete caregiver baseline characteristics are presented in Table 3.

### Home Care Clients’ Admission to Higher Levels of Care

For the primary outcome in Ontario, there was a median follow-up time of 365 days, and the intervention group had an event rate of 5.5 per 10,000 person-days compared with the event rate of 8.0 per 10,000 person-days for the control group (Table 4). The intervention group was associated with a nonsignificant 30% reduction in risk of being unable to return home (HR 0.7, 95% CI 0.3-1.4). For the secondary outcome in Ontario, home care client mortality, there was a median follow-up time of 365 days, and the intervention group had an event rate of 2.9 per 10,000 person-days vs the control group that had an event rate of 2.5 per 10,000 person-days (Table 4). Both groups had similar risk of mortality (HR 1.2, 95% CI 0.4-3.2).

**Table 4:** Unadjusted hazard ratio of primary and secondary outcomes and 95% CIs.

		Ontario				Nova Scotia		
		Control (n=114)	Intervention (n=60)	*P* value	Control (n=94)		Intervention (n=34)	*P* value
**Primary outcome: unable to return home**								
	Events, n (%)	27 (24)	10 (17)	—^a^	8 (9)		≤5	—
	Follow-up in days, median (IQR)	365 (257-365)	365 (334-365)	—	365 (204-365)		365 (200-365)	—
	Event rate per 10,000 person-days	8.0	5.5	—	2.9		3.2	—
	Values, hazard ratio (95% CI)	Reference	0.7 (0.3-1.4)	.30	Reference		1.1 (0.3-3.7)	.90
**Secondary outcome: mortality**								
	Events, n (%)	10 (9)	6 (10)	—	16 (17)		8 (18)	—
	Follow-up in days, median (IQR)	365 (365-365)	365 (365-365)	—	365 (211-365)		365 (214-365)	—
	Event rate per 10,000 person-days	2.5	2.9	—	5.8		6.2	—
	Values, hazard ratio (95% CI)	Reference	1.2 (0.4-3.2)	.80	Reference		1.1 (0.5-2.5)	.90

^a^Not applicable.

For the primary outcome in Nova Scotia, there was a median follow-up of 365 days, and the intervention group had an event rate of 3.2 per 10,000 person-days compared with the event rate of 2.9 per 10,000 person-days for the control group (Table 4). The intervention group was associated with a nonsignificant 10% increase in risk of being unable to return home (HR 1.1, 95% CI 0.3-3.7). For the secondary outcome in Nova Scotia, home care client mortality, there was a median follow-up time of 365 days, and the intervention group had an event rate of 6.2 per 10,000 person-days versus the control group that had an event rate of 5.8 per 10,000 person-days (Table 4). Both groups had similar risk of mortality (HR 1.1, 95% CI 0.5-2.5).

### Health Care Use

#### Ontario Home Care Clients

There was no significant difference in health care use at the 1-year poststudy enrollment for Ontario control home care clients for all services included in the analysis (all *P*>.05). For Ontario intervention home care clients, significantly fewer participants used emergency departments (*P*=.01) at the 1-year poststudy enrollment (24/37, 65%) compared with prestudy enrollment (31/37, 84%). In addition, on average, Ontario intervention home care clients made significantly fewer visits to outpatient services (enrollment: mean 42.2, SD 31.0; after enrollment: mean 32.9, SD 26.0; *P*=.04) and specialist services (enrollment: mean 20.7, SD 25.3; after enrollment: mean 14.6 SD 18.4; *P*=.02). There were no differences in hospital admissions, primary care visits, or prescriptions at the 1-year poststudy enrollment for Ontario intervention home care clients. Complete health care use outcomes for both groups of Ontario home care clients are presented in Table 5.

**Table 5:** Ontario home care client health care use at the prestudy and 1-year poststudy enrollment.

Health care use		Control				Intervention		
		Before enrollment (n=71)	After enrollment (n=71)	*P* value	Before enrollment (n=37)		After enrollment (n=37)	*P* value
**Hospital admissions**								
	Values, n (%)	26 (36.6)	31 (43.7)	.35	21 (56.8)		14 (37.8)	.07
	Values, mean per client (SD)	0.7 (1.2)	0.7 (1.1)	.72	0.9 (1.1)		0.7 (1.0)	.38
**Emergency department visits**								
	Values, n (%)	50 (70.4)	47 (66.2)	.56	31 (83.8)		24 (64.9)	.01
	Values, mean per client (SD)	2.1 (2.4)	2.3 (2.8)	.99	2.5 (2.6)		2.0 (2.2)	.39
**Outpatient physician visits**								
	Values, n (%)	71 (100)	71 (100)	—^a^	37 (100)		37 (100)	—
	Values, mean per client (SD)	37.5 (35.3)	32.1 (28.2)	.35	42.2 (31.0)		32.9 (26.0)	.04
**Primary care visits**								
	Values, n (%)	71 (100)	66-71 (92.9-100)	NR^b^	37 (100)		37 (100)	NR
	Values, mean per client (SD)	14.6 (16.9)	13.0 (11.6)	.86	13.3 (9.5)		12.6 (10.8)	.45
**Specialist visits**								
	Values, n (%)	66-71 (92.9-100)	64 (90.1)	NR	32-37 (86.5-100)		32-37 (86.5-100)	NR
	Values, mean per client (SD)	16.0 (23.4)	13.0 (16.5)	.25	20.7 (25.3)		14.6 (18.4)	.02
**Prescriptions**								
	Values, n (%)	66-71 (92.9-100)	66-71 (92.9-100)	NR	32-37 (86.5-100)		32-37 (86.5-100)	NR
	Values, mean per client (SD)	6.5 (5.3)	6.7 (5.2)	.27	6.4 (4.5)		5.9 (4.4)	.24

^a^Not applicable.

^b^NR: not reported.

#### Nova Scotia Home Care Clients

On average, Nova Scotia home care clients in the control group made significantly fewer visits to primary care at the 1-year poststudy enrollment (enrollment: mean 9.2, SD 5.7; after enrollment: mean 7.8, SD 5.7; *P*=.004). There was no significant difference in health care use at the 1-year poststudy enrollment for Nova Scotia control home care clients for all other services included for the analysis (all *P*>.05). At the 1-year poststudy enrollment, Nova Scotia intervention home care clients averaged significantly more hospitalization visits (enrollment: mean 0.3, SD 0.5; after enrollment: mean 0.6, SD 0.9; *P*=.04) and emergency department visits (enrollment: mean 0.9, SD 1.3; after enrollment: mean 1.5, SD 1.8; *P*=.02) and significantly fewer outpatient services (enrollment: mean 16.6, SD 13.2); after enrollment: mean 13.1, SD 11.4; *P*=.005) and primary care visits (enrollment: 8.5, SD 6.8; after enrollment: mean 6.7, SD 5.5; *P*=.02). There were no differences in prescriptions at the 1-year poststudy enrollment for Nova Scotia intervention home care clients (all *P*>.05). Complete health care use outcomes for both groups of Nova Scotia home care clients are presented in Table 6.

**Table 6:** Nova Scotia home care client health care use at the prestudy and 1-year poststudy enrollment.

Health care use		Control				Intervention		
		Before enrollment (n=71)	After enrollment (=71)	*P* value	Before enrollment (n=34)		After enrollment (n=34)	*P* value
**Hospital admissions**								
	Values, n (%)	19 (26.8)	27 (38)	.10	9 (26.5)		15 (44.1)	.08
	Values, mean per client (SD)	0.3 (0.6)	0.5 (0.8)	.07	0.3 (0.5)		0.6 (0.9)	.04
**Emergency department visits**								
	Values, n (%)	34 (47.9)	39 (54.9)	.28	18 (52.9)		22 (64.7)	.21
	Values, mean per client (SD)	1.2 (1.7)	1.1 (1.5)	.82	0.9 (1.3)		1.5 (1.8)	.02
**Outpatient physician visits**								
	Values, n (%)	71 (100)	71 (100)	—^a^	32 (94.1)		34 (100)	—
	Values, mean per client (SD)	17.4 (9.9)	16.9 (17.2)	.03	16.6 (13.2)		13.1 (11.4)	.005
**Primary care visits**								
	Values, n (%)	71 (100)	69 (97.2)	—	32 (94.1)		33 (97.1)	.56
	Values, mean per client (SD)	9.2 (5.7)	7.8 (5.7)	.004	8.5 (6.8)		6.7 (5.5)	.02
**Specialist visits**								
	Values, n (%)	65 (91.5)	58 (81.7)	.07	26 (76.5)		27 (79.4)	.65
	Values, mean per client (SD)	5.0 (5.7)	5.6 (14.1)	.54	4.4 (5.3)		3.8 (4.1)	.39
**Prescriptions**								
	Values, n (%)	63 (88.7)	65 (91.5)	.53	29 (85.3)		32 (94.1)	.18
	Values, mean per client (SD)	5.3 (3.8)	5.3 (3.8)	.63	5.3 (4.3)		5.3 (3.9)	.62

^a^Not available.

## Discussion

### Primary Outcome of PRM Within Home Care

While our study did not yield statistically significant results regarding the effectiveness of the PRM model in prolonging home stays, the observed trends suggest that technology-assisted aging in place may be a valuable goal for older adults. Despite the underpowered design of our study, the use of the PRM model of care was associated with meaningful, albeit nonstatistically significant, differences between control and intervention groups on the primary outcome (ie, home care clients’ ability to stay at home) in Ontario and Nova Scotia. Specifically, our findings with the Ontario intervention participants trended toward a greater number of home care clients staying home and staying home longer compared with those without PRM in their home. This is similar to the findings of others who have examined the impact of remote monitoring technologies on patient and caregiver well-being [34,35]. The Canadian Expert Panel on Aging highlights the importance of even insignificant delays to admission to LTC [2]. They report that retaining and appropriately supporting older adults at home—even for a month—positively impacts the overall LTC system incapacity or capacity and growing waitlist for care. Furthermore, Canadian older adults’ overwhelmingly prefer to age at home and 11% to 30% of Canadians admitted to LTC could have remained at home and in their communities if adequate home care and community supports were available [36,37].

It is important to highlight several participant characteristics that contextualize the nature of this study’s patient-caregiver dyads when considering participants’ ability to stay at home. The PRM intervention in this study purposefully targeted a select cohort of older adult home care clients (mean age range 77-81 years), many (125/314, 40%) with limited personal resources (ie, education or income) but all who required significant supportive care to remain in their home environment. Home care clients in Ontario were mostly cared for by a spouse living in the same home residence, whereas in Nova Scotia, most older adults lived alone. All older adult home care clients in this study were dealing with significant comorbidities; the most prevalent were hypertension, diabetes, and dementia. In Ontario, about 2 in 5 home care clients were managing dementia, as were approximately 1 in 3 of those in Nova Scotia. This is significant, as previous research demonstrates that older adults (ie, home care clients in this study), especially those with dementia, express a greater quality of life and social connections, less loneliness, and fewer depressive symptoms when living at home compared with institutionalized LTC [38-41]. The health status of home care clients in this study was characteristic of older adults at risk for higher levels of care, with dementia being the greatest predictor of LTC home admissions [2].

### Secondary Outcomes of PRM Within Home Care

What was notable among Ontario intervention home care clients was a significant reduction in their health service use over the course of the study. We found that compared with those receiving usual care, a smaller proportion of Ontario older adult home care clients in the intervention group used emergency departments after 1 year compared with baseline; no such difference was observed in home care clients in the control arm. In addition, Ontario intervention home care clients experienced a significant decrease in the average number of visits to outpatient and specialist services after 1 year, while there were no changes in average visits to these services for home care clients in the control arm. This is an important finding given the *at-risk* health status of older adult home care recipients in the study. Finch et al [35] reported a reduction in health care costs reflective of fewer hospitalizations and emergency visits among “nursing home–eligible” older adults who were monitored by home-based PRM sensors to track their safety (eg, falls) and activities of daily living (eg, time in bed, toilet use, and opening and closing the refrigerator). Consistent with the findings of this study, Finch et al [35] concluded that PRM systems may contribute to a reduction in older adult health service use and associated reductions in health care costs.

However, this was not the case among home care clients in Nova Scotia who received the intervention. In contrast to their Ontario counterparts, Nova Scotia intervention home care clients averaged significantly greater hospital admissions and emergency department visits but significantly fewer outpatient and primary care visits. Discussion with Nova Scotia project partners (health care decision makers) provided some insight into the unanticipated health service use trends of Nova Scotia home care clients in our study. Consistent with our findings, Nova Scotia health system partners reported an observed trend of increased health system use among the Nova Scotia older adult population. The provincial health care decision makers conveyed that emergency services and hospitalization may be a work-around strategy people use to expedite access to LTC services (“skip the line”) and circumvent existing policies governing LTC access. Current policies elevate the LTC priority status of hospitalized at-risk older adults who are on the LTC waitlist and given priority consideration for LTC placement (personal communication, June 2024). Home surveillance of older adults’ behaviors, activities, and habits through PRM technologies by caregivers may also have contributed to increased health service use. Caregivers’ surveillance of older adults’ activities in their home may have illuminated older adults’ need for greater care. In addition, PRM may generate greater insight into older adults’ care needs that may influence (ie, increase) their health service use. This is consistent with pilot study findings of PRM home care services conducted in Nova Scotia (Report of CareLink Evaluation: The Nova Scotia experience, unpublished data, 2015).

Our findings of increased hospital health service use patterns may also be consequential to the 12% to 17% shortfall of primary care service provision within Nova Scotia [42]. With underresourced primary health care services, it may be that Nova Scotians, including complex care older adults needing health care services, would be inclined to access tertiary care services in the absence of primary care services. The Canadian Expert Panel on Aging cautioned that the lack of adequate home care and community-based health care services creates the conditions for premature institutionalization of older adults into LTC. Concernedly, current estimates suggest that up to 1 in 3 Canadians admitted to an LTC home could have remained home with adequate home care services [2]. Avoiding or delaying institutionalized care eases the health system burden particularly in LTC health settings that are perpetually underresourced and ill-equipped to manage the current and pending need for older adult care [43]. An estimated 52,000 Canadian adults are on waiting lists for a placement into an LTC home, while more than 430,000 are estimated to have unmet home care needs, with about 167,000 of them being aged ≥65 years [6,43].

Qualitative research evaluating active remote monitoring and PRM technologies has supported the use of PRM technology as a way to keep at-risk older adults in their home or extend their time at home before requiring institutionalized care [44]. The study, conducted in Nova Scotia, engaged expert key informants including provincial health care policy makers, home care providers, and remote monitoring technology experts to provide their insight into the use of home-based remote monitoring technologies. Key informant participants supported the use of active and PRM technologies as a way to keep complex care older adults at home or extend their time at home. The mitigated risk of adverse events, greater responsiveness to health emergencies (eg, falls), enhanced independence among older adults, and enhanced oversight and greater safety were reported with home monitoring compared with the oversight of care within LTC facilities [44].

### Limitations and Strengths

This study was limited by a restricted sample size included in the primary outcomes analysis. Our study was profoundly affected by the COVID-19 pandemic response; public health measures to mitigate the spread of COVID-19 changed the nature of home care and expectations of family and friend caregivers in the middle of this study’s sampling frame. This impacted the nature of the family caregiving relationships and the nature of support that caregivers were able to provide. The pandemic response also impacted what was considered normal standard of care for home care services due to resources being redirected to other sectors within the publicly funded health care systems in Ontario and Nova Scotia. Despite this, we did observe promising trends in the data related to PRM as a potentially supportive home care strategy among a cohort of older adults with significant and complex care needs. In addition, we acknowledge the unique opportunity to collect data via PRM systems (ie, information from the sensor notifications) regarding older adults’ behaviors within the home setting and the ability to leverage PRM data into large datasets to be analyzed and used to better understand the home care needs of complex care older adults.

Perhaps a strength and limitation of the PRM model of home care for health care systems is the reliance on unpaid caregivers to receive and act on PRM system notifications and not the health care system. Alternatively, the PRM model of home care enhances unpaid caregivers’ responsibility and accountability to the care of their family member.

There was also limited representation of marginalized communities within our study, which limits our understanding of the impact of PRM home care models among culturally diverse populations. Finally, the fact that most caregivers were spouses to the patient participants limited this study’s understanding of how PRM technologies may impact caregivers in the sandwich generation (ie, those individuals caring for older adult parents and children dependents).

### Future Research

There are several opportunities for future research. An economic analysis of the PRM model of care will provide greater insight regarding the implementation of the model of care among health system decision makers. Additional research is needed to fully explore concerns related to home care client, caregiver, and home care provider privacy. Research is needed into the contributions of the PRM model of home care intervention to resolve the challenges of limited health human resources within home care while balancing the health care (physical, emotional, and financial) services given to family caregivers. We also propose an opportunity for data science research in analyzing the aggregated data on older adults’ behaviors to leverage historical tracking of data to support trend analysis and the development of best practices within the home care setting and, importantly, to generate a plan of home care services targeted to the unique needs of complex care older adults. A consideration of this PRM model of care against available DIY or off-the-shelf sensor systems requires investigation. The use of off-the-shelf systems has implications for enhanced digital skills with caregivers and older adults that is not required to the same extent with the PRM model of care in this study. Investigation into older adults’ and caregiver digital health literacy skills will further inform uptake and use of the PRM model of home care. Research into system data visualization—dashboard components for older adult clients and caregivers—would also support informed decision-making and provide insight into user-friendly dashboards. Longitudinal studies with populations with different chronic diseases (eg, cancer and dementia) is also needed to determine PRM model impact.

### Conclusions

The combination of growing waitlists for LTC, limited home care resources, and older adults’ preference to remain in their home as long as possible creates the care context for PRM technologies. These technologies hold promise to support complex care older adults to safely remain in their home and avoid or delay admission to higher levels of care. Home is preferred by most older adults even if their health conditions challenge their ability to live independently; almost all Canadians aged ≥65 years reported their intention to take extraordinary measures to avoid institutionalization in an LTC setting. The PRM model of care in this study relied on unpaid caregivers, not the health care system, to receive and act on PRM notifications. While not statistically significant, the findings of this study demonstrate a trend in favor of PRM to support patients’ aging in place and positive changes in health service use among older adult home care recipients. To appreciate the provincial differences reported in this study, the PRM model of care, as with any health care system intervention, must be considered within the larger health, social, and political context. Further study is required to understand if longer follow-up time allows more effects of PRM on patients’ avoidance of higher levels of care to be detected.

## Appendix

Multimedia Appendix 1 CONSORT eHEALTH checklist (V 1.6.1).

## Abbreviations

HDNS: Health Data Nova Scotia
HR: hazard ratio
ICES: Institute for Clinical Evaluative Sciences
LTC: long-term care
PRCT: pragmatic randomized controlled trial
PRM: passive remote monitoring
